# Supplementary material for: Elucidating the inhibitory mechanism of Zika virus NS2B-NS3 protease with dipeptide inhibitors: Insights from molecular docking and molecular dynamics simulations
Source: PLoS One. 2024 Aug 8;19(8):e0307902. doi: 10.1371/journal.pone.0307902 (PMC11309477; doi:10.1371/journal.pone.0307902)
Supplement: Attachment — Submitted filename: Response to the reviewer.docx [file pone.0307902.s002.docx]

**1^st^ Reviewer**

Reviewer #1: The authors responded to almost all comments and suggestions.

Still proper annotation for the ligand-target interaction is required. The authors annotated for the hydrogen distances only without annotating for the hydrogen bond angles. Hydrogen binding should be presented as both distances and bond angles since hydrogen bond depend on both as to ensure the adequacy of optimum hydrogen bonding

**Answer:** We value your feedback and recommendations about the ligand-target interaction annotation, especially about the need to include hydrogen bond angles. We agree that angle and distance criteria do define hydrogen bonds; these two factors together govern the specificity and strength of the interactions. Our investigation originally concentrated on hydrogen bond distances to give a clear picture of the interactions. However, a more complete and accurate representation of the binding interactions will be provided by including hydrogen bond angles. The angle between the donor atom, the hydrogen atom, and the acceptor atom is the standard way to measure the hydrogen bond angle (D-H...A). This angle usually falls between 150° and 180° for the best hydrogen bonding. The angle criteria filter the hydrogen bonds mentioned in the manuscript. For the sake of visualization, we mentioned the angle criteria in the figure legend and in the manuscript.

**2^nd^ Reviewer**

Thanks you very much for taking your time and accepting our manuscript.
